# Case report: Successful treatment of sebaceous gland hyperplasia using a 1064 nm picosecond laser in an Asian skin type

**DOI:** 10.1016/j.jdcr.2026.07.018

**Published:** 2026-08-05

**Authors:** Sutthinun Wichyanrat

**Affiliations:** Department of Dermatology, Ramathibodi Hospital, Mahidol University, Bangkok, Thailand

**Keywords:** Asian skin type, carbon dioxide laser, photoacoustic, picosecond laser, sebaceous gland hyperplasia

## Introduction

Sebaceous gland hyperplasia is a common benign proliferation of the sebaceous glands. Multiple treatment modalities have been utilized, including electrodessication, ablative carbon dioxide (CO_2_) lasers, pulsed dyed lasers, photodynamic therapy, and isotretinoin medication.^1^^,^^2^ The current picosecond laser has been used for many skin diseases and tattoo removal with favorable therapeutic outcomes.^3^ This case report presents a novel application of picosecond laser for the treatment of sebaceous gland hyperplasia in an Asian skin type, demonstrating successful clinical resolution.

## Case report

A 49 -years-old, Thai female patient with skin type III-IV presented at the outpatient dermatologic and laser department in May 2025, for treatment of sebaceous gland hyperplasia; she required laser photorejuvenation. The patient presented with prominent sebaceous gland hyperplasia on the forehead, characterized by multiple discrete yellowish papules with central umbilicated diameter of 2-5 mm. and height of 1 mm (Fig 1). Initial laser options for her sebaceous gland hyperplasia were a CO_2_ laser and a few sessions of facial photorejuvenation using picosecond laser toning mode.

**Fig 1:**
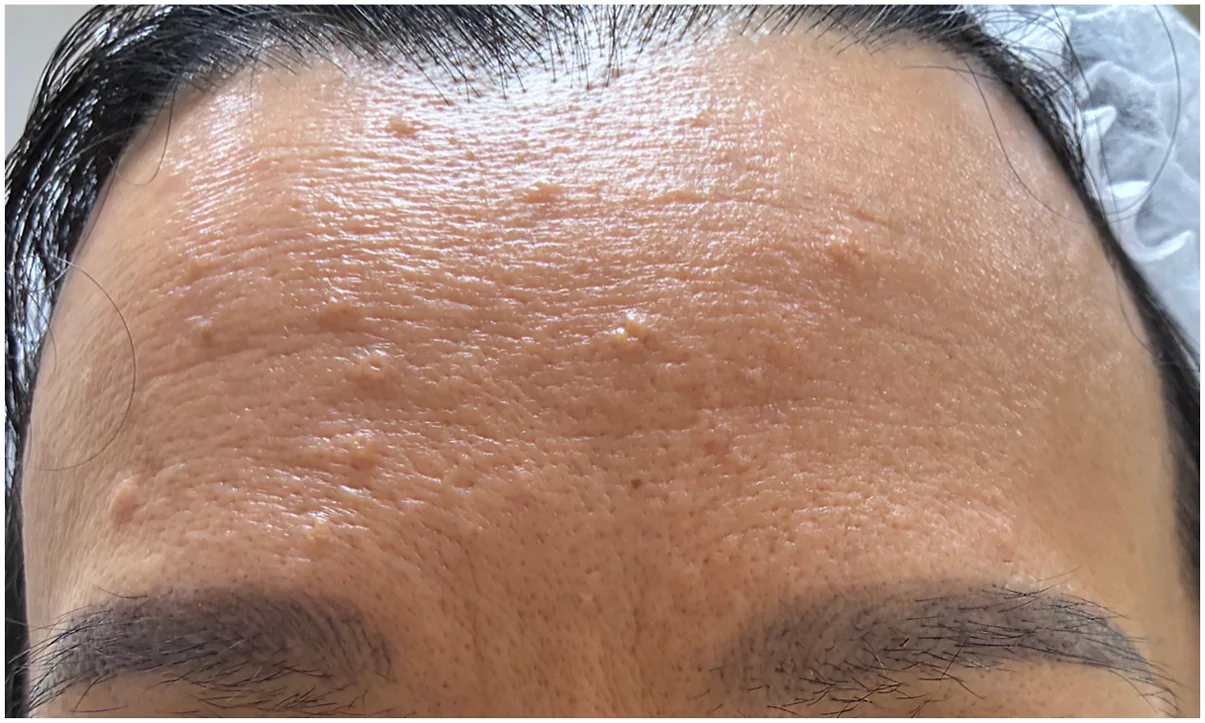
The patient before laser treatment. There are multiple yellowish papules with central umbilication on the forehead.

The potential risk of atrophic scarring following CO2 laser ablation, particularly on the forehead, was explicitly discussed with the patient. The patient expressed a strong preference for minimizing potential scarring in the forehead area as a primary concern. A therapeutic decision was made to utilize a 1064 nm picosecond laser for the treatment of sebaceous gland hyperplasia. The applied parameters included a spot size of 3 mm, fluence of 4.3 J/cm^2^, and a frequency of 1-2 Hz (Picoway,Candela). Additionally, a test spot using a CO2 laser (AcuPulse™,Luminis), 0.7 Watts, super pulse, continuous mode; was performed on a single lesion for comparative analysis. (Fig 2).

**Fig 2:**
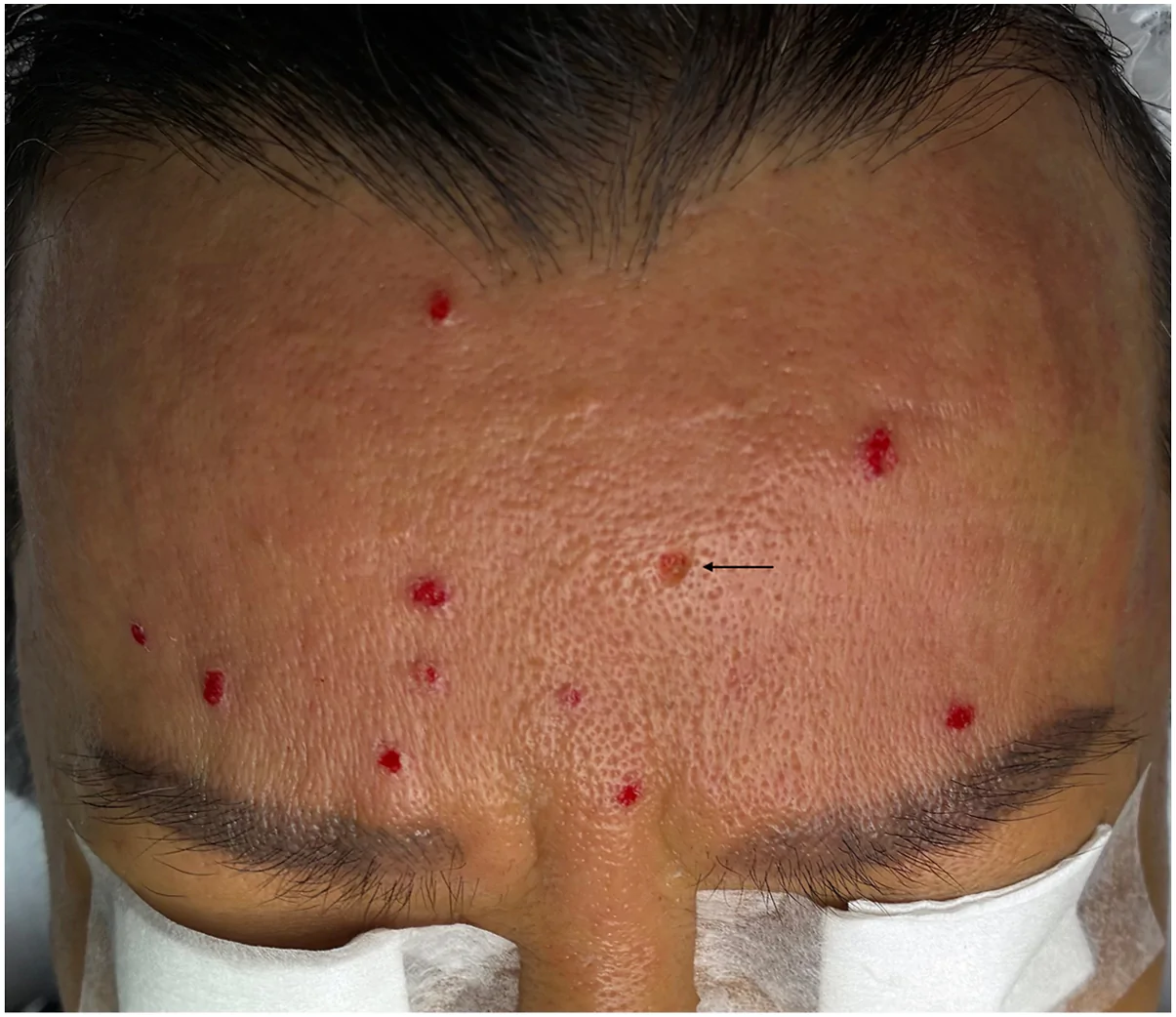
Immediately after picosecond laser and CO_2_ test spot. The end point of the picosecond laser was epidermal whitening. After a few minutes, punctate bleeding was observed. After CO_2_ laser test spot, a single lesion (*arrowhead*) was completely ablated. *CO*_*2*_, Carbon dioxide.

Clinical results demonstrated a 75% to 100% improvement in the lesions following picosecond laser therapy (Fig 3). However, multiple treatment sessions are required to achieve the desired near-total clearance of the sebaceous gland hyperplasia (three-session interval every 4 weeks). The complication of picosecond laser was mild post-inflammatory hyperpigmentation which was treated with topical 20% azelaic acid cream, and the condition improved after 2 months. While the CO2 laser test spot achieved complete destruction of the sebaceous gland hyperplasia in a single session, it resulted in the formation of atrophic scarring; thus, the best laser to treat sebaceous gland hyperplasia for the patient was picosecond laser.

**Fig 3:**
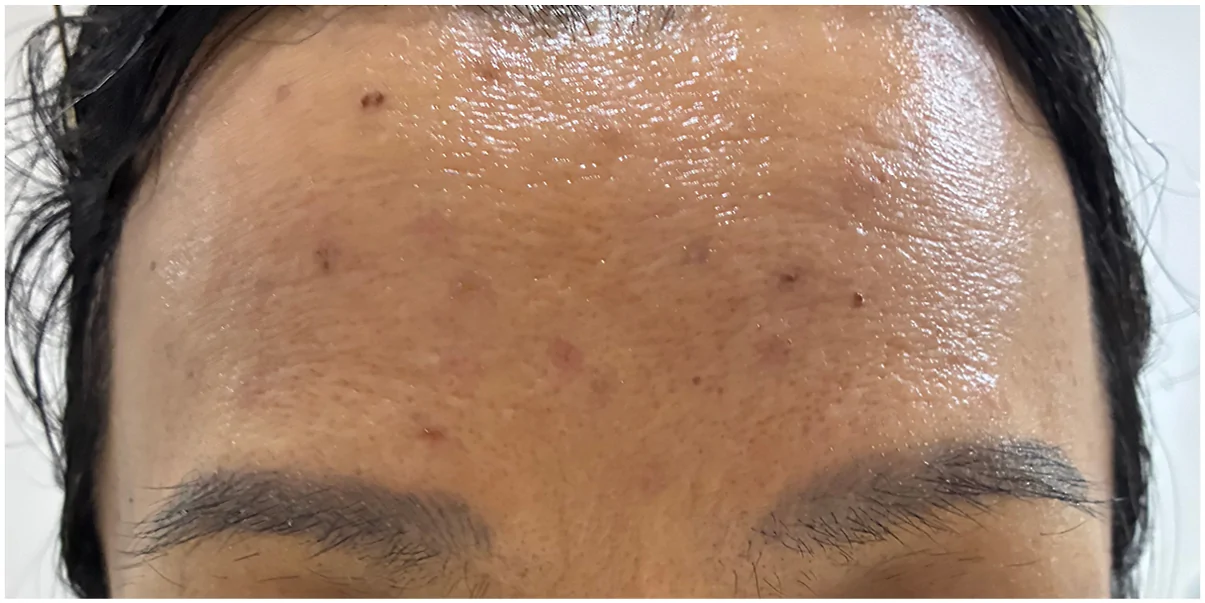
Follow up picture after 1 month; Lesion treated with a picosecond laser show 50% to 75% improvement in sebaceous gland hyperplasia with post-inflammatory hyperpigmentation. A CO_2_ treated lesion shows mild atrophic scar. *CO*_2_, Carbon dioxide.

## Discussion

Previous studies reported the use of Q-switched Nd:YAG nanosecond and picosecond lasers for the treatment of xanthelasma palpebrarum.^4^, ^5^, ^6^ However, clinical data regarding the application of picosecond lasers, specifically for sebaceous gland hyperplasia remains limited. A picosecond laser was administered to sebaceous gland hyperplasia lesions, targeting a photoacoustic endpoint characterized by immediate epidermal whitening and punctate bleeding. Picosecond lasers facilitate deep dermal penetration, triggering multimodal mechanisms to achieve the selective destruction of sebaceous gland hyperplasia by unknown mechanisms and may be attributed to the synergistic effect of selective photothermolysis and photoacoustic. It targets multiple chromophores, including lipids, hemoglobin, and melanin. Furthermore, the photoacoustic effect may specifically address age-related endogenous pigments (such as lipofuscin)^7^ within the dermal environment, leading to cellular rejuvenation and tissue remodeling.

This case report describes an Asian skin type that might have more melanin in the sebaceous gland and cause complications such as post-inflammatory hyperpigmentation. Picosecond laser treatment for sebaceous gland hyperplasia reduced atrophic scarring, although sequential sessions are required to achieve complete lesion resolution. Post-treatment care for picosecond laser involves application of white petrolatum or topical antibiotic ointments for 7 days, representing a less intensive regimen than full-thickness ablation required after CO2 laser therapy. At the 9-month follow-up, the patient exhibited no clinical recurrence of sebaceous gland hyperplasia in the treated areas. (Fig 4).

**Fig 4:**
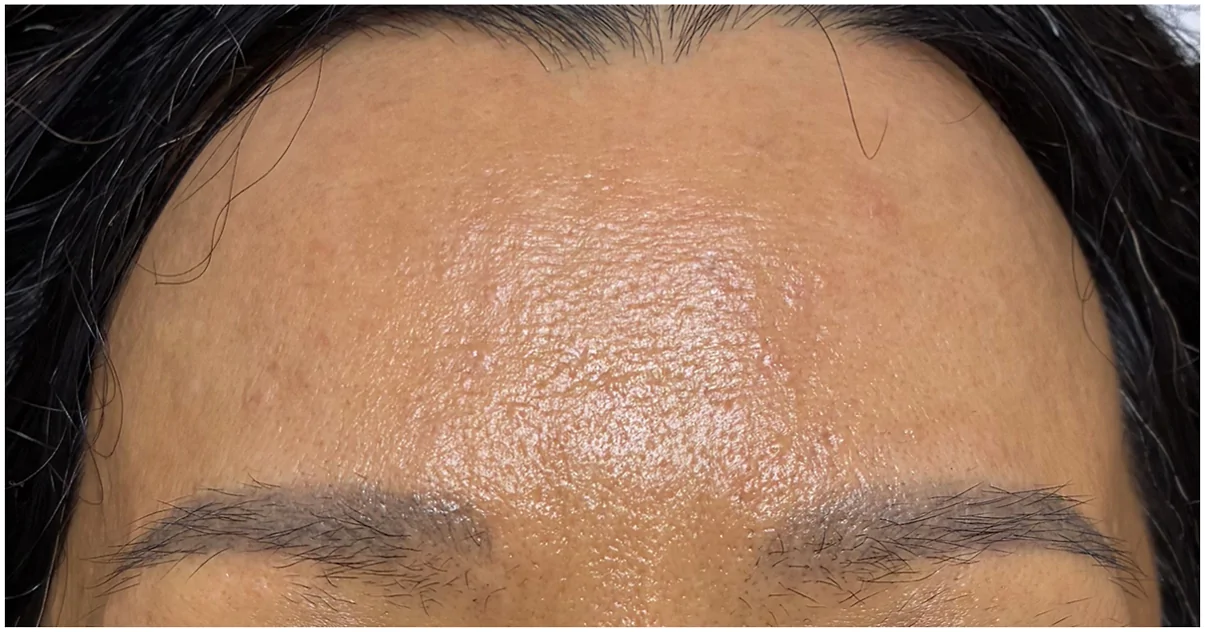
Follow-up after 9-month treatment. Post-picosecond 1064-nm laser for three sessions every 4 weeks. The improvement was 90% to 100% with no recurrence of sebaceous gland hyperplasia. CO_2_-treated test spot still exhibited a mildly atrophic scar. *CO*_*2*_, Carbon dioxide.

## Conclusion

We report a successful case of sebaceous gland hyperplasia on the forehead of an Asian patient treated with a 1064-nm picosecond laser. The clinical outcome demonstrated superior esthetic results with a significantly lower incidence of atrophic scarring compared with traditional CO_2_ ablative laser therapy. Further randomized controlled trials with larger sample sizes are warranted to validated the finding of this study and establish standardized treatment protocols.

## Conflicts of interest

None disclosed.
